# Lipopolysaccharide-Induced Loss of Cultured Rat Myenteric Neurons - Role of AMP-Activated Protein Kinase

**DOI:** 10.1371/journal.pone.0114044

**Published:** 2014-12-02

**Authors:** Ulrikke Voss, Eva Ekblad

**Affiliations:** Department of Experimental Medical Science, Lund University, Lund, Sweden; University of California, Los Angeles, United States of America

## Abstract

**Objective:**

Intestinal barrier function is vital for homeostasis. Conditions where the mucosal barrier is compromised lead to increased plasma content of lipopolysaccharide (LPS). LPS acts on Toll-like receptor 4 (TLR4) and initiates cellular inflammatory responses. TLR4 receptors have been identified on enteric neurons and LPS exposure causes neuronal loss, counteracted by vasoactive intestinal peptide (VIP), by unknown mechanisms. In addition AMP activated protein kinase (AMPK) stimulation causes loss of enteric neurons. This study investigated a possible role of AMPK activation in LPS-induced neuronal loss.

**Design:**

Primary cultures of myenteric neurons isolated from rat small intestine were used. Cultures were treated with LPS (0.2–20 µg/mL) with and without TAK1-inhibitor (5Z)-7-Oxozeaenol (10^−6^ M) or AMPK inhibitor compound C (10^−5^ M). AMPK-induced neuronal loss was verified treating cultures with three different AMPK activators, AICAR (10^−4^−3×10^−3^ M), metformin (0.2–20 µg/mL) and A-769662 (10^−5^−3×10^−4^ M) with or without the presence of compound C (10^−5^ M). Upstream activation of AMPK-induced neuronal loss was tested by treating cultures with AICAR (10^−3^ M) in the presence of TAK1 inhibitor (5Z)-7-Oxozeaenol (10^−6^ M). Neuronal survival and relative numbers of neurons immunoreactive (IR) for VIP were evaluated using immunocytochemistry.

**Results:**

LPS caused a concentration dependent loss of neurons. All AMPK activators induced loss of myenteric neurons in a concentration dependent manner. LPS-, AICAR- and metformin-,but not A-769662-, induced neuronal losses were inhibited by presence of compound C. LPS, AICAR or metformin exposure increased the relative number of VIP-IR neurons; co-treatment with (5Z)-7-Oxozeaenol or compound C reversed the relative increase in VIP-IR neurons induced by LPS. (5Z)-7-Oxozeaenol, compound C or A-769662 did not *per se* change neuronal survival or relative numbers of VIP-IR neurons.

**Conclusion:**

AMPK activation mimics LPS-induced loss of cultured myenteric neurons and LPS-induced neuronal loss is counteracted by TAK1 and AMPK inhibition. This suggests enteric neuroimmune interactions involving AMPK regulation.

## Introduction

The gastrointestinal (GI) tract comprises the body's largest surface to the outside environment. It is vital for nutrient uptake and contains the human microbiome, consisting of more than 100 trillion microorganisms with different properties. [1], [2] The importance of a functional barrier is highlighted in conditions such as post-operative ileus, functional bowel disorders and obesity, where a compromised barrier causes inflammatory responses of different severity. [3]–[5] Increased permeability of the intestinal barrier commonly leads to increased plasma levels of lipopolysaccharide (LPS), a major component of gram negative bacteria membranes. LPS binds to toll like receptor 4 (TLR4) and initiates an inflammatory response. [6] The transforming growth factor-β-activated kinase 1 (TAK1) is an important regulator of cellular responses initiated by environmental stress. [7] As a downstream effector-molecule common to e.g. TLR4-, interleukin-1- and tumor necrosis factor-receptor stimulation it is closely linked to the innate immune response. [6], [7]

A key player in regulating digestive, in particular intestinal, functions is the enteric nervous system (ENS). The ENS is optimally situated within and along the digestive tract where it is pivotal in regulating intestinal motility, blood flow and secretion. Dysregulation of ENS causes GI symptoms and jeopardizes intestinal barrier integrity. LPS exposure *in vitro* has previously been shown to cause loss of porcine and rat enteric neurons, probably through TLR4 activation since this receptors is expressed on a subpopulation of enteric neurons. [8], [9] Furthermore, vasoactive intestinal peptide (VIP) has been highlighted as being protective in the response to LPS mediated TLR4 activation. It reduces LPS-induced inflammation and enteric neuronal loss. [9], [10]

The evolutionarily well conserved AMP-activated protein kinase (AMPK) is central in cellular metabolism and energy regulation. It acts as a metabolic switch, conveying cellular and hormonal responses both short and long term. AMPK is a heterotrimeric complex consisting of a catalytic α subunit and two regulatory β/γ subunits. It is activated by allosteric binding of AMP to domains on the γ subunit and phosphorylation of Thr172 on the α subunit. Depending on the combination of subunit isoforms AMPK can display different signalling properties. [11], [12] Studies investigating AMPK in inflammation have suggested diverse roles. In microglia cultures and cell lines LPS has been shown to activate AMPK thereby mediating cytokine release. [13]–[15] In macrophages, however, AMPK activation inhibits LPS-induced activation, causing reduced inflammation. [16], [17] AMPK activation, using AICAR has even been shown to reduce the pro-inflammatory cytokine response in TNBS-induced colitis and LPS-induced lung injury. [16], [17] Current study using pharmacologic *in vitro* experimentation was designed to investigate mechanisms underlying LPS-induced enteric neuronal loss.

## Methods

### Ethics statement

Procedures were approved by the regional Malmö/Lund committee for experimental animal ethics, under the Swedish board of Agriculture, (diary number M152-12). Animals were used in accordance with the European Community Council Directive (2010/63/EU) and the Swedish Animal Welfare Act (SFS 1988∶534).

### Animals and tissue preparations

Female Sprauge-Dawley rats (Charles River, DE), (n = 23, 130–180 g) were used. Primary myenteric neuronal cultures from the small intestine were prepared as described previously. [18] From each animal 6 culture plates of 8 wells (BD Bioscience, SE) were prepared, animals were never pooled. The resulting cultures containing both myenteric neurons and enteric glia were grown 4 days in medium (neurobasal A, containing 10% fetal bovine serum, 0.5 mM L-glutamine, 50 U/mL penicillin and 50 µg/mL streptomycin, all from Life Technologies, SE). Fresh medium containing applicable experimental test agents was then added and incubation for an additional 4 day period followed. Control wells were cultured in parallel. Cells were fixed in Stefaninis fixative, rinsed in Tyrode solution, frozen, thawed and subjected to immunocytochemistry. [18]

### Pharmacological agents and experimental set-ups

Stock solutions of 5-amino-β-D-ribofuranosyl-imidazole-4-carboxamide (AICAR, Sigma-Aldrich, SE), 6,7-Dihydro-4-hydroxy-3-(2'-hydroxy[1,1'-biphenyl]-4-yl)-6-oxo-thieno[2,3-*b*]pyridine-5-carbonitrile (A-769662, Tocris, UK), metformin hydrochloride (Cayman Chemicals, UK), 6-[4-(2-Piperidin-1-ylethoxy)phenyl]-3-pyridin-4-ylpyrazolo[1,5-a]pyrimidine (compound C, Sigma-Aldrich, SE), (5Z)-7-Oxozeaenol (Tocris Bioscience, UK) and Escherichia coli serotype O111∶B4 derived LPS (Sigma-Aldrich, SE) were prepared according to manufactures recommendations, aliquoted and stored at 20°C.

Various sets of experiments were performed. Incubations were for 4 days. Cultures were exposed to 1. LPS (0.2–20 µg/mL) with or without (5Z)-7-Oxozeaenol (10^−6^ M) or compound C (10^−5^ M), 2. (5Z)-7-Oxozeaenol (10^−7^–10^−5^ M), 3. compound C (3×10^−7^–3×10^−5^ M), 4. AICAR (10^−4^–3×10^−3^ M), A-769662 (10^−5^–3×10^−4^ M) or metformin (10^−7^–10^−3^ M), 5. AICAR (10^−3^ M), A-769662 (10^−4^ M) or metformin (10^−4^ M) together with compound C (10^−5^ M), 6. AICAR (10^−3^ M), metformin (10^−4^ M) or LPS (20 µg/mL) together with (5Z)-7-Oxozeaenol (10^−6^ M). Controls were always run in parallel.

### Immunocytochemistry

For details on primary and secondary antibodies see table 1. All antibodies were diluted in phosphate buffered saline containing 0.25% Triton X-100 and 0.25% BSA. Double immunolabelling of cultures was performed by overnight incubation in moist chambers at 4°C with a mixture of primary antibodies. [9] Secondary antibodies were mixed and incubated 1 h at RT. Hoechst (Life Technologies, SE) cell nuclei counter staining was performed according to manufacturer's protocol. Mounting was in PBS∶glycerol 1∶1 followed by fluorescence microscopy (Olympus BX43, LRI, SE) with appropriate filter setting.

**Table 1:** Overview of primary and secondary antibodies used in immunocytochemistry.

Raised against	Dilution	Code	Source	Host	References
Human neuronal protein, (HuC/HuD)	1∶600	A21272	Life Technologies, SE	Mouse	[18], [19]
Human gene product 9.5, (PGP 9.5), purified human brain	1∶1.200	RA95101	Ultraclone, UK	Rabbit	[19]
Vasoactive intestinal peptide, purified porcine	1∶1200	7852	Euro-Diagnostica, SE	Rabbit	[20]
Mouse IgG	1∶1.000	115-545-166	Jackson Lab Inc, USA	Goat	
Rabbit IgG	1∶1.000	711-585-152	Jackson Lab Inc, USA	Donkey	

### Neuronal analyses

Neuronal survival was estimated according to previously described protocol. [18], [21] In brief, neuronal survival after exposure to the various treatments was calculated by counting the total number of HuC/HuD-immunoreactive (IR) neurons in the entire culture well (69 mm^2^) and expressed as percentage of the number of total neurons in the control well run in parallel (% neuronal survival of control). Relative number of neurons immunoreactive for VIP was estimated from cultures double immunolabeled for HuC/HuD and VIP. Results were expressed as the percentage of HuC/HuD-IR neurons also positive for VIP (% VIP-IR neurons).

### Statistical analyses

Data are presented as means ± SEM and analyzed by GraphPad Prism (GraphPad Software Inc, USA). Every experimental group contain n = 3−57 repeats from a minimum of 3 different animals. Statistical significances were determined using one-way-analysis-of-variance followed by Dunnet's post hoc test towards controls. A confidence level of 95% was considered significant.

## Results

All investigations included a 4 day pre-culture period followed by 4 days in treatment conditions. Untreated controls were always run in parallel and neuronal survival was calculated and expressed as percentage of control. Control wells displayed a large number (3.3±0.03 neurons/mm^2^, n = 57, average seeding density 226 neurons/well) of evenly distributed neurons, with a dense varicose fibre network.

### LPS effect on myenteric neurons

**Figure 1:**
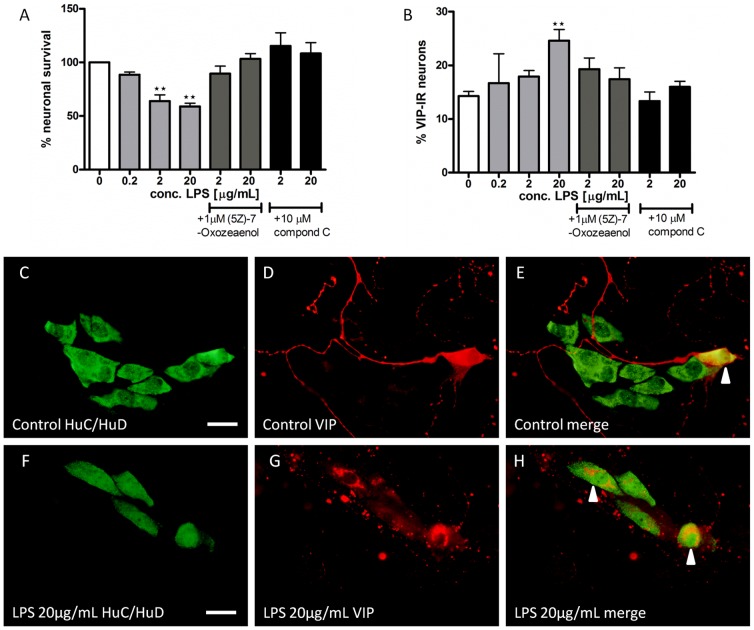
LPS- induced effects on survival and relative numbers of VIP-immunoreactive (IR) myenteric neurons are attenuated by simultaneous addition of (5Z)-7-Oxozeaenol or compound C. A. shows neuronal survival, expressed as % of controls, after exposure to LPS (0.2–20 µg/mL) and after simultaneous exposure to LPS (2–20 µg/mL) and either TAK-1 inhibitor (5Z)-7-Oxozeaenol (10^−6^ M) or compound C (10^−5^ M). Nerve cell bodies were identified using immunostaining against HuC/HuD. Exposure of LPS reduces neuronal survival in a concentration dependent manner. Both (5Z)-7-Oxozeaenol and compound C protect against LPS induced neuronal loss. B. shows the relative numbers of VIP-IR neurons, expressed in percentage of HuC/HuD-IR neurons, after exposure to LPS (0.2–20 µg/mL) and after simultaneous exposure to LPS (2–20 µg/mL) and either TAK-1 inhibitor (5Z)-7-Oxozeaenol (10^−6^ M) or compound C (10^−5^ M). LPS increases the relative numbers of VIP-IR neurons compared to controls. This effect is blocked by simultaneous exposure with (5Z)-7-Oxozeaenol (10^−6^ M) or compound C (10^−5^ M). Data are expressed as mean ± SEM, ** p<0.01, n = 3–30. C–H representative micrographs of cultured myenteric neurons double immunolabled with (C and F) HuC/HuD and (D and G) VIP and (E and H) merged. C–E control culture, F–H LPS (20 µg/mL) treated culture. Arrowheads in E and H mark neurons IR for both Huc/HuD and VIP. Bar represents 20 µm.

Presence of LPS (0.2–20 µg/mL) caused a concentration dependent loss of cultured myenteric neurons. At highest concentrations tested (20 µg/mL) survival was reduced by 45%, figure 1A. Presence of LPS also increased the relative number of VIP-IR neurons, in a concentration dependent manner, figure 1B. Representable micrographs of control and LPS treated cultures are shown in figure 1C–H.

### TAK1 inhibitor (5Z)-7-Oxozeaenol exposure

**Figure 2:**
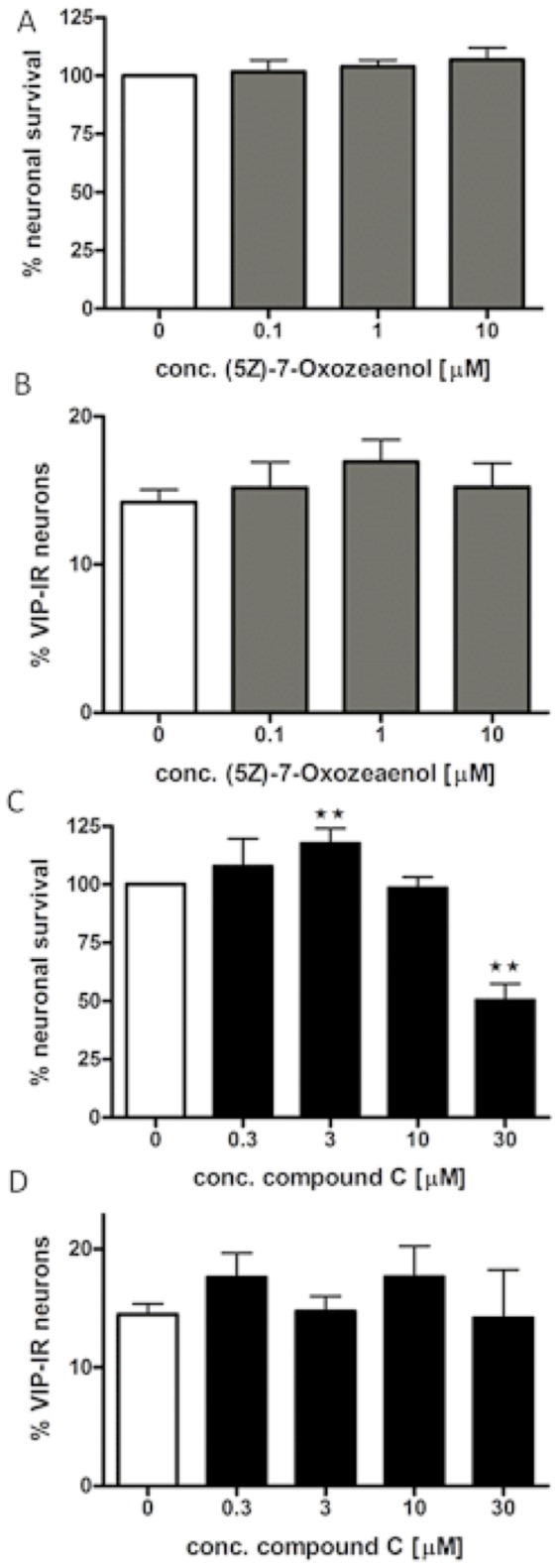
(5Z)-7-Oxozeaenol- and compound C-induced effects on survival and relative numbers of VIP-immunoreactive (IR) neurons. A. shows neuronal survival, expressed as % of controls, after exposure to TAK-1 inhibitor (5Z)-7-Oxozeaenol (10^−7^–10^−5^ M). Nerve cell bodies were identified using immunostaining against HuC/HuD. Exposure to (5Z)-7-Oxozeaenol does not change neuronal survival in cultures. B. shows that the relative number of VIP-IR neurons expressed as percentage of HuC/HuD-IR are unchanged after (5Z)-7-Oxozeaenol (10^−7^–10^−5^ M), compared to control. C. shows neuronal survival, expressed as % of controls, after exposure to compound C (3×10^−7^–3×10^−5^ M). Exposure to compound C displays a biphasic pharmacological profile with low concentrations (3×10^−6^ M) increasing and high concentrations (3×10^−5^) decreasing neuronal survival. D. shows that the relative numbers of VIP-IR neurons are unchanged after exposure to compound C (3×10^−7^–3×10^−5^ M), compared to controls. Data are expressed as mean ± SEM, ** p<0.01, n = 5–20.

(5Z)-7-Oxozeaenol is a selective and irreversible inhibitor of TAK1. [22] Presence of TAK1 inhibitor (10^−7^–10^−5^ M) did not alter neuronal survival, (figure 2A) or the relative number of VIP-IR neurons (figure 2B) compared to control. Based on this pharmacological profile and profiles described in, [22] 10^−6^ M was chosen as working concentration in the following experiments.

### Compound C has a biphasic pharmacological profile on neuronal survival

Compound C is demonstrated as a competitive AMPK antagonist. [11] Exposing cultures to low concentrations (3×10^−7^–3×10^−6^ M) of compound C caused a slight increase in neuronal survival, compared to controls. Higher concentrations (10^−5^–3×10^−5^ M) resulted in a concentration dependent reduction in neuronal survival, figure 2C. Compound C (3×10^−7^–3×10^−5^ M) exposure caused no change in the relative number of VIP-IR neurons, figure 2D. Based on the here shown pharmacological profile of compound C, 10^−5^ M was chosen as working concentration in the following experiments.

### LPS-induced neuronal loss and relative increase in VIP-IR neurons are reversed by (5Z)-7-Oxozeaenol or compound C

Simultaneous exposure of LPS (20 µg/mL) and (5Z)-7-Oxozeaenol (10^−6^ M) or compound C (10^−5^ M) did not change either neuronal survival or the relative number of VIP-IR neurons within the treated cultures compared to control, figure 1. Thus, presence of (5Z)-7-Oxozeaenol or compound C abolished the previously described LPS-induced myenteric neuronal loss and relative increase in the number of VIP-IR neurons.

### AMPK activators mimic the effect of LPS on myenteric neurons

**Figure 3:**
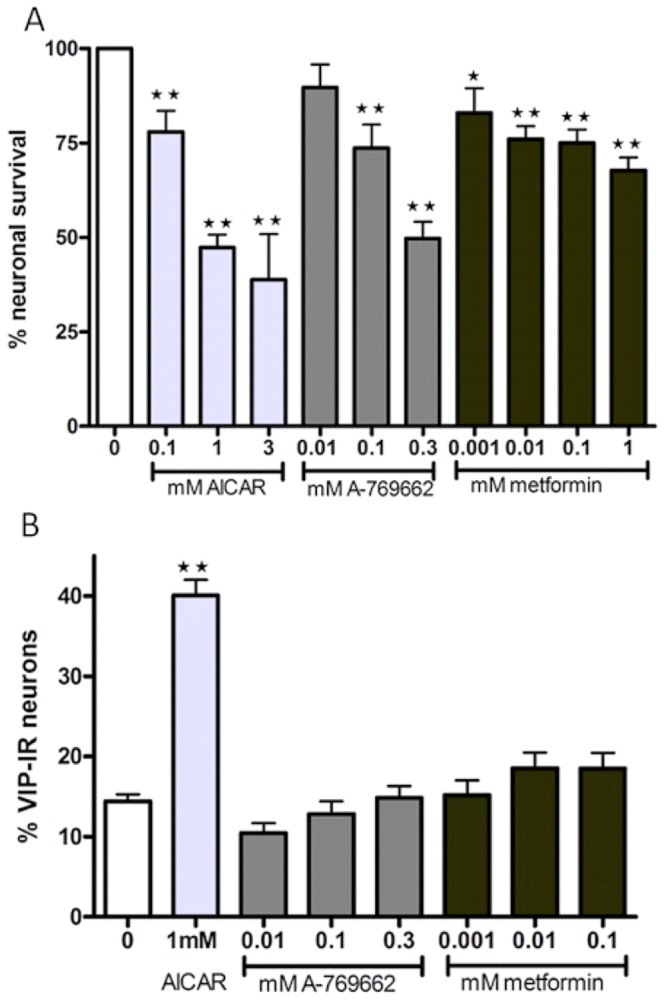
Effects of AMPK activation on survival and relative numbers of VIP-immunoreactive (IR) myenteric neurons. A. Neuronal survival, expressed as % of controls, after 4 days of exposure to AICAR (10^−4^–3×10^−3^ M), A-769662 (10^−5^–3×10^−4^ M) or metformin (10^−6^–10^−3^ M). Nerve cell bodies were identified using immunostaining against HuC/HuD. AICAR, A-769662 and metformin induced a concentration dependent loss of neurons. B. shows the relative numbers of VIP-IR neurons, expressed in percentage of HuC/HuD-IR neurons, after exposure to AICAR (10^−3^ M), A-769662 (10^−5^–3×10^−4^ M) or metformin (10^−6^–10^−3^ M). AICAR increases the relative numbers of VIP-IR neurons while A-769662 and metformin does not, compared to control. Data expressed as mean ± SEM, ** p<0.01, n = 4–24.

AICAR acts as an AMP analogue and A-769662 acts as a reversible activator of the AMPK β/γ subunits. [23], [24] Metformin is suggested to interact with the γ-AMPK subunit causing a conformational change, [25] or to activate AMPK indirect through inhibition of the respiratory chain altering cellular energy status. [26] Exposing cultures to increasing concentrations of AICAR (10^−4^–3×10^−3^ M), A-769662 (10^−5^–3×10^−4^ M) or metformin (10^−6^–10^−3^ M) caused a concentration dependent loss of neurons, figure 3A. A-769662 (10^−5^–3×10^−4^ M) or metformin (10^−6^–10^−4^ M) exposure did not change the relative proportion of neurons IR for VIP, while AICAR (10^−3^ M) exposure caused an increase, figure 3B.

### Compound C protects against AICAR- and metformin-, but not A-769662-, induced neuronal loss

**Figure 4:**
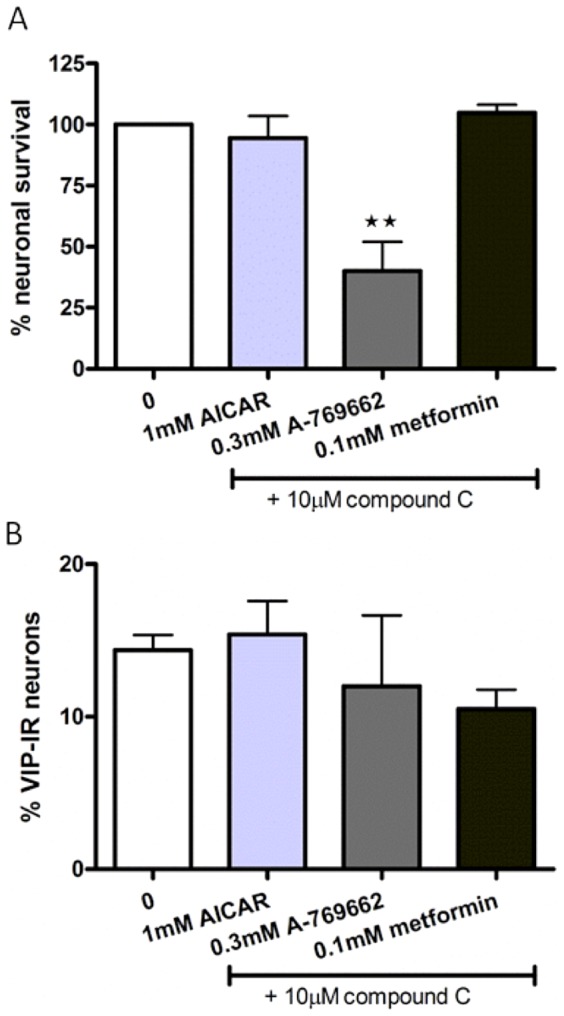
Effects of compound C on AMPK activation on survival and relative numbers of VIP-immunoreactive (IR) myenteric neurons. A Neuronal survival, expressed as % of controls, after exposure to AICAR (10^−3^ M), A-769662 (3×10^−4^ M) or metformin (10^−4^ M) and compound C (10^−5^ M). Nerve cell bodies were identified using immunostaining against HuC/HuD. Compound C protects against AICAR- and metformin, but not A-769662-, induced neuronal loss. B. The relative numbers of VIP-IR neurons, expressed in percentage of HuC/HuD-IR neurons, are unchanged after simultaneous exposure to AICAR (10^-3^ M), A-769662 (3×10^−4^ M) or metformin (10^−4^ M) and compound C (10^−5^ M), compared to controls. Data expressed as mean ± SEM, ** p<0.01, n = 3–12.

Simultaneous exposure of AICAR (10^−3^ M) or metformin (10^−4^ M) and compound C (10^−5^ M) did not affect neuronal survival nor the relative number of VIP-IR neurons within the cultures, thus, compound C abolished the previously described AICAR- and metformin-induced myenteric neuronal losses and AICAR-induced relative increase in the number of VIP-IR neurons. On the other hand, simultaneous exposure of A-769662 (3×10^−4^ M) and compound C (10^−5^ M) caused loss of myenteric neurons in the same magnitude as A-769662 alone. Results are summarized in figure 4.

### (5Z)-7-Oxoaeaenol does not inhibit AICAR- or metformin-induced neuronal loss

**Figure 5:**
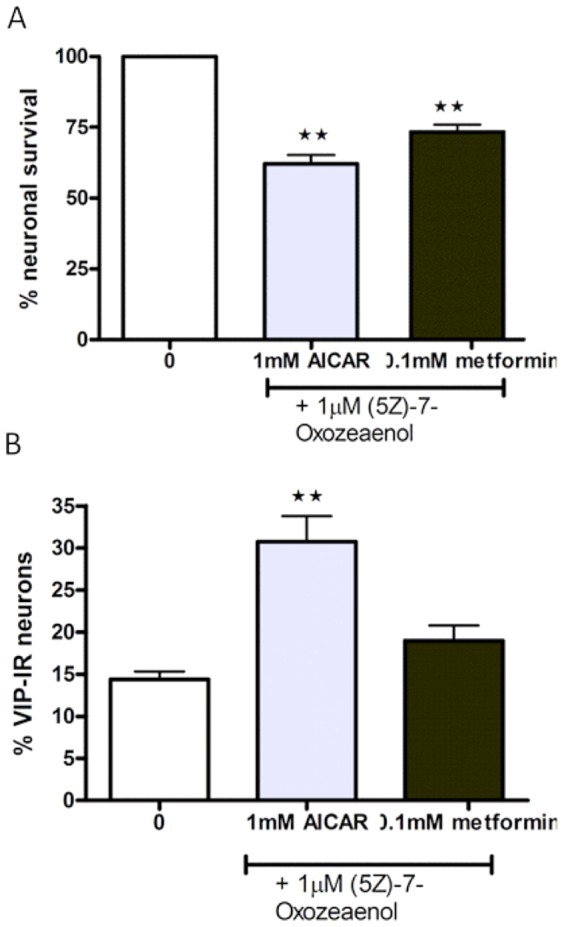
Effects of (5Z)-7-Oxozeaenol on AMPK activation on survival and relative numbers of VIP-immunoreactive (IR) myenteric neurons. A. neuronal survival, expressed as % of controls, after exposure to AICAR (10^−3^ M) or metformin (10^−4^ M) and TAK-1 inhibitor (5Z)-7-Oxozeaenol (10^−6^ M). Nerve cell bodies were identified using immunostaining against HuC/HuD. (5Z)-7-Oxozeaenol does not protect against either AICAR- or metformin-induced neuronal loss. B. (5Z)-7-Oxozeaenol (10^−5^ M) are unable to reduce the AICAR (10^−3^ M)-induced relative numbers of VIP-IR neurons, expressed in percentage of HuC/HuD-IR neurons. VIP-IR after simultaneous exposure of 5Z)-7-Oxozeaenol (10^−5^ M) and metformin (10^−4^ M) are unchanged compared to controls. Data expressed as mean ± SEM, ** p<0.01, n = 7–14.

Simultaneous exposure of AICAR (10^−3^ M) or metformin (10^−4^ M) and the TAK1 inhibitor (5Z)-7-Oxozeaenol (10^−6^ M) caused loss of cultured myenteric neurons in the same magnitude as AICAR (10^−3^ M) or metformin (10^−4^ M) alone. Presence of (5Z)-7-Oxozeaenol (10^−6^ M) did not affect AICAR-induced up-regulation of the relative number of VIP-IR neurons. Results are summarized in figure 5.

## Discussion

Current study re-affirmed previous findings showing that Escherichia coli derived LPS induces neuronal loss in primary cultures of adult myenteric neurons. [8], [9] The novel findings presented here are that presence of the AMPK inhibitor compound C protects myenteric neurons against LPS-induced neuronal loss and that, by using three independent AMPK activators, AMPK activation was found to mimic LPS exposure in that it induced loss of cultured myenteric neurons. The link between LPS-induced TLR4 stimulation and AMPK activation was further strengthened by the finding that TAK1 is involved. This since the presence of TAK1 inhibitor (5Z)-7-Oxozeaenol protected against LPS-induced neuronal loss but not against AICAR-induced neuronal loss.

### LPS-induced AMPK activation

Elevated fat and energy intake increases plasma LPS levels in both patients and mice, leading to increased expressions of TLR4 and exacerbation of inflammation. [3], [27] Current study revealed LPS-induced neuronal loss to be mediated by AMPK activation. The reduced neuronal survival evident after LPS exposure was blocked by simultaneous presence of the AMPK inhibitor compound C. These findings are corroborated by previous findings on primary microglia cultures and cell lines in which LPS was found to cause AMPK activation and cytokine release, through TLR4 activation. [13]–[15] The link between TLR4 activation and AMPK activation is suggested to be TAK1, a key player in regulating the cellular response to environmental and cytokine-induced stress. TAK1 is able to regulate several transcription factors including IKKβ, p38/JNK and NF-kB, [7], [28] and has previously been shown to mediate AMPK activation. [29]–[31] Enteric neurons are sensitive to LPS and express TLR4. [9] The significance of such expression is suggested to be regulation of the innate tolerance response to commensal bacteria in the intestine. [9] This ensures a balanced immune response with respect to luminal content and homeostasis. Supporting this, is the finding that TLR4^-/-^ mice display a delayed transit time. [32] In contrast to previous, [8], [9] and current findings, LPS has been reported to increase survival of enteric neuronal cultures through TLR4 signalling. [32] This discrepancy is suggested due to differences in LPS concentration; low concentrations are protective while high ones are deleterious. It may also be due to differences in TLR4 expression and LPS sensitivity between embryonic (gut not colonized with microbiota), [32] and adult [8], [9] enteric neurons. Also LPS exposure duration and potency are probably of importance. *In vivo* the adaptive immune response primed by bacterial exposure is, in the healthy intestine with a functional barrier, probably mediated by low mucosal permeability and hence low LPS concentrations. TLR4 activation may under such healthy conditions promote maintenance of neurons to ensure survival. In conditions of compromised barrier function and permeability, systemic LPS reach higher concentrations. This change in LPS concentration may cause hyper-activation of TLR4 and imminent dysregulation of the adaptive immune response eventually leading to death. Another intriguing possibility that should be considered is the origin of LPS. Though many of the gram negative bacterial strains are the source of adverse infections some are considered beneficial. [33], [34] The molecular part of LPS interacting with TLR4 is the membrane anchoring region, called lipid A. [35] This region consists of a phosphorylated N-acetylglucosamine dimer with a varying amount of saturated fatty acid tails in both tail number and chain length attached. [35] The ability of this region to interact with the TLR4 complex elicits a fine-tuned immune response. [35], [36] The properties of the LPS variants used in previous studies investigating LPS-induced effects on ENS need to be taken into consideration when interpreting and comparing results.

### AMPK activation in neurons

The results show AMPK activation to be detrimental to myenteric neurons; this was confirmed using three different AMPK activators, AICAR, metformin and A-769662. AMPK-induced neuronal loss has previously been suggested to occur in enteric, [37] as well as in central [38] neurons. In the CNS compound C was found to protect against stroke-induced neuronal loss. [38] The suggested mechanism behind was that compound C attenuated the AMPK activated energy processes causeing increased cellular stress in already compromised neurons. A balanced AMPK response is necessary in models of ischemic stress, which induces a transient increase of AMPK activation and autophagy. This is believed to be part of the protective response that, if unbalanced, leads to cell death. [39] Further, AMPK activation has, in neuronal cell lines, been shown to decrease neurite growth causing loss of contact and eventually neurodegeneration. [40] These studies are well in line with the here presented data, showing that activation of AMPK is detrimental to enteric neuronal survival. Interestingly TAK1 inhibition, like AMPK inhibition, is suggested to mediate neuroprotection also of central neurons after ischemic or traumatic brain injury. [41], [42]

The here noted difference in sensitivity to compound C by AICAR, metformin and A-769662 induced neuronal is likely explained by their molecular targets. AICAR is an AMP analogue activating AMPK in a the same manner as AMP; an allosteric conformational change and activation. [23] Compound C is a competitive inhibitor binding to the same ligand site as AMP and AICAR. [11] On the other hand, A-769662 has a different AMPK activation mechanism involving ligand site separate from AMP and depends on the presence of the β1 subunit. [24], [43] These differences in ligand binding and activation patterns may well explain why compound C blocks AICAR, but not A-769662, induced neuronal loss. Metformin- like AICAR-induced loss was blocked by compound C. Whether this, in analogy with AICAR, can be explained by a competitive binding is more uncertain. The mechanism by which metformin activate AMPK is currently debated. Both a direct interaction with AMPK, [25] as well as several possible indirect mechanisms have been suggested involving both AMP dependent and independent mechanism. Metformin is a commonly used drug in the treatment of type 2-diabetes and its positive effects on this disease may be ascribed to other signalling pathways than AMPK activation. [26] It is worth noting that in the present study while using a concentration within the reported physiological range, [47], [48] metformin was used as an AMPK activator.

### LPS- and AMPK activation-induced VIP expression

The LPS-induced increase in the relative proportion of VIP immunoreactive myenteric neurons has previously been reported, together with the finding that exogenous VIP protects against LPS-induced neuronal loss. [8], [9] These reports further speculated if the physiological significance of an increased number of VIP-IR neurons was due to VIP's immunmodulatory effects, or due to its general neuroprotective properties. [9], [49], [50] Current study showed LPS and AICAR, but not metformin or A-769662, to increase the relative numbers of VIP immunoreactive neurons, an increase that could be attenuated by simultaneous exposure to compound C. The differences between AICAR, metformin and A-769662 in evoking an increased relative number of VIP-IR neurons are enigmatic. AICAR has been shown to have intracellular secondary activation targets. [51], [52] This may provide one explanation since A-769662, which is believed to be a more precise AMPK activator, [24] did not up-regulate VIP. The unresolved mechanism of metformin's activation of AMPK makes interpretation of the metformin effect on VIP-IR neurons harder to discuss. However, as compound C was able to reverse both the LPS- and AICAR-induced upregulation of VIP-IR neurons the possibility of a direct AMPK-induced effect can't be completely disregarded. In muscle cells AMPK is able to phosphorylate the transcription factor cAMP responsive element binding protein (CREB) at the same site as protein kinase A. [53] AMPK is thereby, through phosphorylation events, able to cause an increase in CREB-dependent transcription leading to increased VIP expression. [54] The mechanism behind the lack of VIP up-regulation by A-769662 and metformin needs further investigation taking into account the diverse regulatory factors both on transcriptional and translational levels. The rationale behind such AMPK-induced neurotransmitter up-regulation needs further investigations. VIP is a neuroimmunopeptide that besides acting as an inhibitory neurotransmitter in ENS also is able to regulate TLR expression. [10] VIP has been shown to down regulate LPS-induced TLR4 expression and subsequent the inflammatory response. [10] It can be speculated that LPS- and AMPK activation-induced VIP up-regulation is part of a protective neuronal response that may, as previously described, play a role in modulating the innate immune-response to commensal bacteria or inflammation. [9]

## Conclusion

LPS exposure and activation of AMPK cause neuronal loss of cultured myenteric neurons. LPS-induced neuronal loss is blocked by the presence of compound C and therefore suggested to be executed through activation of AMPK. The link between LPS and AMPK is suggested to be TAK1 acting downstream of TLR4 and upstream of AMPK. This suggestion is supported by the finding that (5Z)-7-Oxozeaenol is able to attenuate LPS- but not AICAR- or metformin-induced neuronal loss. Further, both LPS and AICAR cause significant increase in the relative numbers of VIP-IR neurons. The rationale behind upregulation of VIP is suggested assigned both to activate adaptive innate neuroimmune-responses to intestinal commensal bacteria and to provide neuroprotection.

## Supporting Information

Dataset S1 **Neuronal survival of control and relative VIP-IR data.**
(XLSX)
